# Intermittent Boluses of Local Anesthetic Through Quadratus Lumborum Catheters for Analgesia in a Living Donor Hepatectomy

**DOI:** 10.1155/2019/1246256

**Published:** 2019-12-23

**Authors:** Shelly B. Borden, Molly K. Groose, Eric R. Simon, Aaron S. Hess, Kristopher M. Schroeder

**Affiliations:** ^1^Anesthesiologist, Assistant Professor, Department of Anesthesiology, University of Wisconsin School of Medicine and Public Health, Madison, WI, USA; ^2^Anesthesiology Resident, Department of Anesthesiology, University of Wisconsin School of Medicine and Public Health, Madison, WI, USA; ^3^Anesthesiology Fellow, Department of Anesthesiology, University of Wisconsin School of Medicine and Public Health, Madison, WI, USA; ^4^Anesthesiologist, Associate Professor, Department of Anesthesiology, University of Wisconsin, School of Medicine and Public Health, Madison, WI, USA

## Abstract

The demand for liver transplants in the United States far exceeds the supply of organs. As need has increased, so has use of living donors. Coagulopathy and various side effects often preclude the use of neuraxial regional techniques and opioids for postoperative analgesia in patients with large “J” incisions. Here, we present a 25-year-old male undergoing a living donor hepatectomy who received quadratus lumborum catheters placed percutaneously after closure of incision and prior to emergence to provide excellent analgesia and a viable opioid-sparing approach. Quadratus lumborum catheters are a safe option for a multimodal, opioid-sparing approach to analgesia.

## 1. Introduction:

The demand for liver transplants in the United States far exceeds the supply of organs. As need has increased, so has use of living donors. In 1998, 92 (2.0%) of 4,519 liver transplants were from living donors, in 2018 that number was 401 (4.9%) of 8,250 [1]. Patients who volunteer to donate often do so with altruistic intentions. Highest-quality safety and analgesia is essential in order to respect this altruism and encourage future donors. Postoperative pain can have deleterious effects including prolonged rehabilitation, development of chronic pain, and reduction in quality of life [2]. Improvements in pain control can reduce the hospital length of stay, reduce hospital costs, and increase patient satisfaction [2].

Opioids have been a cornerstone in postoperative pain management in patients undergoing abdominal surgery. However, in addition to the classic opioid side effects including sedation, nausea, pruritus, and constipation, many opioids are extensively metabolized by the liver. This can lead to difficulties with titration and the potential for unintentional overdose in a patient with a small native liver remnant after undergoing the extensive hepatectomy required for living liver donation. Therefore, regional anesthesia techniques have been employed for living donor hepatectomy. Thoracic epidural placement has long been the gold standard for analgesia. However, some studies reveal postoperative coagulopathy as a result of liver resection; this can increase the risk of neuraxial hematoma and subsequent nerve injury [3, 4]. Further, preoperative thrombosis prophylaxis ordered by surgeons (in this case, enoxaparin) precludes neuraxial administration of medications such as intrathecal morphine. Subcostal transversus abdominis plane (TAP) blocks have also been described as options for regional anesthesia, but single injection techniques with standard local anesthetics rarely extend analgesia beyond 12–24 hours [5]. Paravertebral catheters have been utilized, but the risk of pneumothorax as well as coagulopathy concerns often prevent its use [6]. Liposomal bupivacaine may provide extended analgesia when utilized for abdominal wall blockade, but this medication is expensive and not widely available [7]. Few case reports but no true randomized controlled trials confer its benefit in fascial plane blocks, particularly the quadratus lumborum block. Goals for treatment of a living liver donor are to employ a multimodal analgesia and opioid-sparing approach to postoperative analgesia. In this case report, these goals were achieved by utilizing the quadratus lumborum (QL) block [8].

The purpose of this case report is to describe the use of an ultrasound-guided placement of bilateral quadratus lumborum catheters (QLC) in a living donor liver transplantation. Written informed consent for treatment and patient's approval for the publication of results were obtained.

## 2. Case Description

A 25-year-old male (American Society of Anesthesiology physical status I; height 179 cm, weight 88.4 kg; body mass index 27.6 kg/m^2^) presented as a living liver donor. He was otherwise healthy. Preoperative vital signs included an oxygen saturation (SpO_2_) of 99%, noninvasive blood pressure 120/73 mmHg, and heart rate 56 bpm. The patient had a baseline Numerical Pain Rating Scale (NRS) score of 0/10.

The patient was seen by the anesthesiologist in the preoperative assessment clinic the day before surgery. Consent was obtained for quadratus lumborum catheters. Preoperatively on the day of surgery, the patient received gabapentin 300 mg per os and enoxaparin 40 mg subcutaneous injection (per surgeon's request for thrombosis prophylaxis). General anesthesia was induced with midazolam, lidocaine, fentanyl, and propofol, with rocuronium for neuromuscular blockade. Donor right hepatectomy was uneventful and successful. Intraoperatively, during the nine-hour procedure, the patient received intermittent fentanyl boluses (total dose 400 mcg) and a single bolus of ketamine 25 mg prior to incision followed by a ketamine infusion at 10 mg/hr, which was continued for 48 hours postoperatively. Upon initiation of surgical closure, the patient received ketorolac 15 mg IV, acetaminophen 1000 mg IV, and hydromorphone 0.6 mg IV.

**Figure 1:**
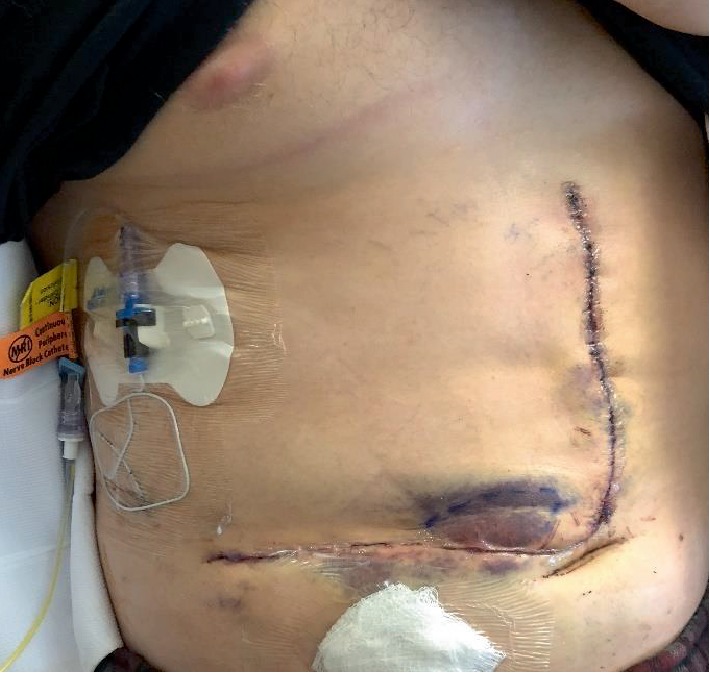
Patient with “J” incision and right-sided quadratus lumborum catheter. Left-sided catheter not shown in figure.

**Figure 2:**
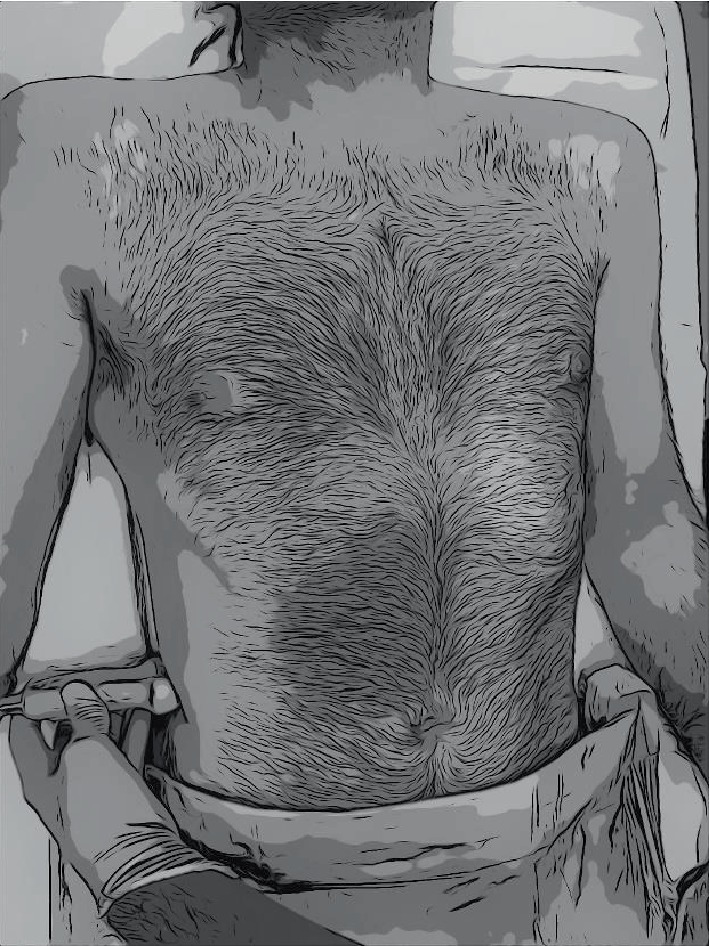
Placement of linear ultrasound transducer on the posterior aspect to allow visualization of posterior aponeurosis of the transversus abdominis muscle.

**Figure 3:**
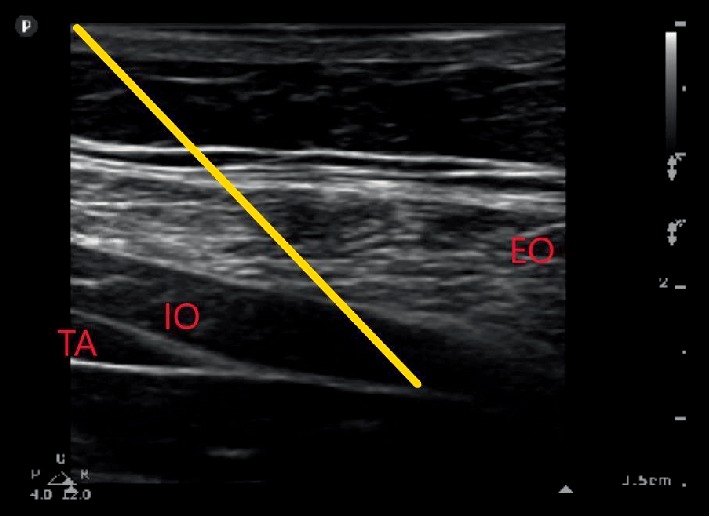
Ultrasound image obtained to place nerve catheters. EO = external oblique muscle. IO = internal oblique muscle. TA = transversus abdominis muscle. Yellow line depicts trajectory of needle into posterior aponeurosis of transversus abdominis muscle.

After closure of the right abdominal “J” incision and prior to emergence, ultrasound-guided QLC were placed bilaterally (Figure 1). A linear ultrasound transducer was placed in the anterior axillary line and moved posteriorly until the posterior aponeurosis of the transversus abdominis muscle became visible (Figure 2). In this lateral QL block (QL type 1), a 17-gauge Tuohy needle was utilized to thread a nerve catheter into the fascial plane. The Tuohy was inserted from the anterior end of the transducer and advanced until this aponeurosis was penetrated (Figure 3). Dissection with sterile saline confirmed the location, and after threading the catheter, 5 mL of 0.5% bupivacaine with 1 : 200,000 epinephrine was injected through the catheter between the aponeurosis and the transversalis fascia at the lateral margin of the QL muscle. Good local anesthetic spread was visualized on ultrasound. Following emergence from anesthesia, the patient endorsed decreased cutaneous sensation to cold most densely at T8-11 on the right anterior abdomen and T7-11 on the left anterior abdomen. Recovery room opioid requirements included 0.4 mg of hydromorphone. Postoperative analgesic needs were comanaged by the surgical and anesthesia services. The surgical team managed breakthrough analgesic (opioid) medications, while the Acute Pain Service managed the QL and ketamine infusions. QL catheter infusions consisted of programmed intermittent boluses of 0.1% ropivacaine at 10 ml/hr on each side. This was initiated at one hour after arrival into the post anesthesia care unit. There was no basal infusion and no patient-controlled local anesthetic boluses. The ketamine infusion continued at 10 mg/hr for 48 hours, then it was stopped. Scheduled ketorolac 15 mg IV every 8 hours was continued until POD 3 and acetaminophen 500 mg every 6 hours and gabapentin 300 mg three times daily were administered until discharge. Tramadol was available for breakthrough pain, but was never used. On postoperative day three, the QL catheters were removed. NRS pain scores (0–10) were recorded every four hours and ranged from 2–4 at rest and 2–4 with activity. From postoperative day one until discharge on postoperative day six, no opioids were administered to the patient. Ultimately, the patient was very satisfied with his level of analgesia throughout the postoperative course.

## 3. Discussion

Over the past two decades, Enhanced Recovery After Surgery (ERAS) programs have successfully been applied to many surgical specialties following the significant benefits initially seen in elective colorectal surgery, including decreased length of stay and decreased postoperative complications [9]. A common theme of ERAS protocols is to minimize opioid use, as opioids are associated with multiple short-term side effects including nausea, vomiting, ileus, urinary retention, and delirium, all of which can delay hospital discharge. High compliance with multimodal, nonopioid based ERAS protocols significantly reduces opioid requirements and leads to improved outcomes [10].

Recently, various ERAS protocols have been proposed for living donor hepatectomy, including recommendations for multimodal analgesia with opioid sparing techniques, such as thoracic epidural analgesia [11], intrathecal morphine [12], transversus abdominis planus blocks [13], or local injection of liposomal bupivacaine [14]. Although neuraxial techniques are often used for living donor hepatectomy, there are unresolved issues regarding its safety in this patient population. Despite having normal liver function before surgery, the majority of donors develop a coagulopathy postoperatively, with a mean peak INR of 1.9 on postoperative day two, and a mean nadir platelet count of 150,000/mm^3^ on postoperative day two or three [14]. Therefore, until the safety of epidural techniques can be clearly demonstrated in these patients, many anesthesiologists prefer a less invasive regional anesthetic technique.

Abdominal fascial plane blocks may be safer to perform in these patients who are likely to develop a postoperative coagulopathy. The classic lateral transversus abdominis plane (TAP) block reliably provides analgesia in the T10-12 dermatomes, however is unlikely to provide analgesia for the upper abdomen, and the “J” incision often extends as cephalad as T6.

Since TAP blockade is limited to somatic anesthesia of the abdominal wall, is highly dependent on interfascial spread, and may not cover the most cephalad aspect of a “J” incision, a QL block may be superior. A QL block is a more recently described fascial plane block where local anesthetic is injected adjacent to the quadratus lumborum muscle with the goal of anesthetizing the thoracolumbar nerves [15]. The QL block can result in extensive sensory blockade (T7-L2) and the successful use of a QL block has been published for many types of surgeries including breast, abdomen, hip, and lower extremity [15]. Here, we present the first published case report of the successful use of bilateral QL catheters as part of an opioid-sparing ERAS protocol for a living donor hepatectomy.
